# Effects of Rapid Maxillary Expander and Delaire Mask Treatment on Airway Sagittal Dimensions in Pediatric Patients Affected by Class III Malocclusion and Obstructive Sleep Apnea Syndrome

**DOI:** 10.3390/life13030673

**Published:** 2023-03-01

**Authors:** Sara Caruso, Emanuela Lisciotto, Silvia Caruso, Alessandra Marino, Fabiana Fiasca, Marco Buttarazzi, David Sarzi Amadè, Melania Evangelisti, Antonella Mattei, Roberto Gatto

**Affiliations:** 1 Department MeSVA, University of L’Aquila, 67100 L’Aquila, Italy; 2 Department of Neurosciences, Mental Health and Sensory Organs, Suicide Prevention Center Sant’Andrea Hospital, Sapienza University of Rome, 00185 Roma, Italy; 3 Independent Researchers, 50134 Firenze, Italy

**Keywords:** obstructive sleep apnea syndrome, class III malocclusion, Delaire mask, rapid maxillary expansion

## Abstract

Obstructive sleep apnea syndrome (OSAS) is a sleep-related breathing disorder that is very common in pediatric patients. In the literature, there are very few studies concerning the association between OSAS and class III malocclusion in children. The use of a rapid maxillary expander (RME) in association with the Delaire mask is a common treatment protocol for class III malocclusion. The aim of this work was to evaluate the cephalometric variations of upper airway dimensions and OSA-related clinical conditions after orthodontic treatment with an RME and the Delaire mask, as recorded in pediatric patients with a class III malocclusion who were affected by OSAS. In this preliminary study, 14 pediatric patients with mixed dentition, aged between 6 and 10 years, were selected. All patients were treated with an RME and the Delaire mask. Pre- and post-treatment cephalometric radiographs were traced, analyzed, and compared. The results demonstrated a significant increase in the upper airway linear measurements and the nasopharyngeal and oropharyngeal dimensions (*p* ≤ 0.05). This increase creates an improvement in airway patency and in OSAS-related clinical conditions. The use of the RME in association with the Delaire mask can be effective in the treatment of pediatric patients with a class III malocclusion who are affected by OSAS.

## 1. Introduction

Sleep-related breathing disorders in pediatric patients are caused by partial or complete obstruction of the upper airways, which can cause episodes of hypopnea or apnea with subsequent repercussions on pulmonary ventilation, oxygenation, and sleeping quality [1]. Obstructive sleep apnea syndrome (OSAS) is characterized by day and night symptoms and signs such as snoring, sleep apnea, drowsiness, decreased concentration and memory ability, enuresis, nocturia, increased blood pressure, heart rate changes, night sweats, irritability, dry mouth, and growth retardation. OSAS-related clinical conditions can also accentuate comorbidities in frail pediatric patients [2]. Numerous studies in the scientific literature show that the most frequent cause of OSAS in childhood is represented by adenotonsillar hypertrophy [3] but other risk factors have also been highlighted, such as rhinitis, allergies, snoring, obesity, craniofacial anomalies, and neuromuscular diseases [4,5]. The most frequent craniofacial alterations reported in children with OSAS are retrognathia, midface hypoplasia, contraction of the upper jaw, relative macroglossia, and anterior open bite [6]. Among these craniofacial anomalies, it has been highlighted that hypoplasia of the maxilla and/or mandible may promote the development of sleep-related breathing disorders [7,8]. Two recent systematic reviews evidenced the association between maxillomandibular discrepancy and OSA, suggesting that children with OSA have more skeletal Class II characteristics and a dolichofacial mandibular growth direction compared to normal children [2,3,4].

The clinical diagnosis of OSAS in children is very sensitive but is not specific to the diagnosis of OSAS when compared to nocturnal polysomnography (PSG) [9]. Clinical history and physical examination only serve the purpose of identifying the subjects in whom clinicians will have to carry out diagnostic instrumental tests [10]. PSG is the current gold standard diagnostic test for OSA, and the apnea-hypopnea index (AHI, which records the number of obstructive and mixed apneas and hypopneas per hour of total sleep time) is the PSG parameter most commonly reported to discriminate the presence and severity of OSA [11]. According to the most recent national guidelines, a diagnosis of OSAS is established when the AHI is >1 ev/h [12]. However, PSG, as the current confirmatory procedure, is cost-prohibitive and is not widely available. Using screening questionnaires for OSA is particularly useful when clinicians need to determine the presence of OSA. Employing specific and accredited questionnaires for the clinical diagnosis of this condition can also offer a good reliability index [11].

Orthopedic therapy is effective in restoring the correct relationship between the maxillary bone and the mandibular bone and between the dental arches. The efficacy of treatment using rapid palate expansion is amply demonstrated in the literature [13,14,15] in children with OSAS and malocclusion. This treatment can operate on the transversal maxillary deficit and extend the base of the nasal cavities, with an increase in nasopharyngeal and oropharyngeal air space and, consequently, can create an improvement in the patency of the upper respiratory tract [16,17]. In patients with class III malocclusion, the overgrowth of the jaw is often accompanied by hypoplasia of the maxillary bone [18]. Despite this connection, in the literature, there is little evidence of the association between class III malocclusion and sleep-related breathing disorders; therefore, there are no validated therapy protocols regarding this clinical condition.

Therapy with an RME and a Delaire mask is one of the most common orthopedic treatment protocols for Class III malocclusions. Among the variety of treatments proposed, we have adopted a therapeutic protocol for Class III malocclusion by maxillary correction. The excellent results reported in experimental works have influenced the clinical adoption of such an approach [19,20]. 

The use of both orthopedic devices has a synergistic effect in promoting the growth of the maxilla in the sagittal and transverse dimensions, while simultaneously limiting mandibular overgrowth [21,22]. 

Nevertheless, very few studies have investigated the use of an RME in association with the protraction of the maxilla to improve the nasopharyngeal and oropharyngeal airway dimensions [23].

Based on these findings, the objective of this work was to evaluate the effects of this protocol treatment via cephalometric radiographs analysis, in terms of upper airway patency, and the improvement of OSAS-related clinical conditions.

## 2. Materials and Methods

### 2.1. The Sample

For this study, we selected 14 pediatric patients (6 males and 8 females) with mixed dentition and aged between 6 and 10 years. At the time of diagnosis, all patients had a class III malocclusion and clinical conditions that are associated with OSAS. Before treatment, informed consent was required from the parents of each child. The patients were treated at the Pediatric Sleep Center (Sant’Andrea Hospital, Rome, Italy). The inclusion criteria for the cases were: patients with signs, symptoms, and clinical history of OSAS and PSG diagnosis (mild, moderate, or severe OSAS), patients aged between 6–10 years, with mixed dentition and, in addition, semiotics and cephalometric standards of class III malocclusion. Moreover, patients with erupted central permanent incisors before the start of orthodontic therapy and those patients who had received treatment with a palate expander according to the same method described below were included. Finally, we included patients for whom lateral teleradiographs were available before and after treatment performed using the same technique, and with whom it was possible to evaluate the patency of the airways.

The exclusion criteria applied to those patients previously treated by surgical therapy for sleep-related breathing disorders (adenoidectomy, tonsillectomy, or adenotonsillectomy) and patients who were taking drugs that could alter respiratory function. Obese patients were also excluded because that may influence the onset of malocclusions [24]. Patients with the characteristics of class I and class II malocclusions, as well as patients with craniofacial anomalies, malformations and/or genetic pathologies, and systemic pathologies, which can alter respiratory functions, were also excluded.

A diagnosis of class III malocclusion was made by clinical examination and confirmed by radiographic evaluation. The patients considered in the study group had clinical conditions associated with sleep-related breathing disorders, a medical history of OSAS, or a previous OSAS diagnosis. Data collection regarding OSAS-related clinical conditions was carried out using clinical examination and specific questionnaires [25]. Afterward, the diagnosis of OSAS was established when the AHI index was >1 ev/h, according to AASM guidelines [12]. For each patient, skeletal, dental, and pharyngeal cephalometric variables were measured and analyzed, then considered on the cephalometric tracings at time T0 before treatment and at T1, at the end of treatment. Cephalometric analysis is useful as a screening test to characterize skeletal and soft tissue relationships in children experiencing OSAS [26]. As a prospective analysis with 95% of power and a level of significance of alpha = 0.05, the sample size was calculated to need at least 9 participants.

For the cephalometric evaluation, both angular and linear variables were measured, as reported in Table 1.

### 2.2. Orthodontic Assessment and Orthopedic Therapy

All patients were treated with an RME. The rapid palatal expansion devices that were used were fixed, with a central expansion screw and cemented bands on the second deciduous molars and with hooked arms extended to the palatal surfaces of the canines and first deciduous molars. All patients were treated with the same expansion protocol. After cementation, the RME was activated. The rapid expansion protocol performed involved two rounds/day for 15 days. After the expansion time, the RME was blocked and kept in the mouth for an average duration of 12 months. After blocking the RME, patients were treated with a Delaire mask. Extraoral protraction with elastics was performed, directly connected to the cemented RME. Patients had to wear the mask all night and for two hours during the day [21]. The mask treatment had an average duration of 6 months, with a two-month follow-up until the end of the treatment. The overall treatment had an average duration of 18 months.

### 2.3. Statistical Analysis

Descriptive statistics were compiled to characterize the patient population. Continuous variables (age, dental, and skeletal and oropharyngeal variables) were expressed as median and interquartile ranges (IQRs) since the data were not normally distributed (the Shapiro–Wilk test); categorical variables (gender, clinical conditions associated with respiratory sleep disorders, and the presence of mixed dentition) were expressed as absolute and percentage frequencies. The Wilcoxon signed-rank test was used to compare dental, skeletal, and oropharyngeal variables at T0 and T1. Statistical analyses were performed with STATA/IC 15.1 For all statistical tests, significance was two-sided and was set at *p* ≤ 0.05.

## 3. Results

A total of 14 patients were included in the study with a median age of 8 years old (IQR 7–9). More than half were females (*n* = 8/14, 57%). Before treatment, 64.29% (*n* = 9/14) were affected by apnea, 50% (*n* = 7/14) by rhinitis and allergy, more than half (*n* = 8/14, 57.14%) had adenotonsillar hypertrophy, and only 14.29% (*n* = 2/12) of patients were affected by snoring. No patient was obese. The clinical conditions associated with sleep-related breathing disorders in the study group are summarized in Table 2, showing their frequency in the present sample. 

The analysis of the pre-treatment and post-treatment cephalometric variables led to the following results.

At baseline, the median values of the angles 1^MAX, 1^MAND, and 1s^1i were 101° (IQR 95–109) 84.5° (IQR 81–89), and 145° (IQR 139–154), respectively (*p* ≤ 0.05). After the orthodontic treatment, the values of the same angles were significantly varied: the first two were increased, 1^MAX 114° (IQR 104–116) and 1^MAND 90° (IQR 87–94), while the third decreased to 1s^1i 131° (IQR123–136). In addition, we found a variation in the sagittal position of the maxilla and mandible in terms of the SNA, SNB, and ANB angles, which varied from 78.5° (IQR 77–81), *p* ≤ 0.05), 77° (IQR 76–82), and 2.5° (IQR 1–5) at T0 to 81.5° (IQR 78–87), 77.5° (IQR 75–85), and 3° (IQR 2–4), respectively, at T1. Moreover, before treatment, the median values of 1s-N-FH and 1i-N-FH angles were 41.15 mm (IQR 37.4–45.5) and 39.5 mm (IQR 37–43), respectively; at the end of the treatment, we recorded 44.75 mm (IQR 42–52.5) and 45 mm (IQR 41–49.5), respectively.

A comparison of the dental and skeletal variables analyzed at times T0 and T1 is shown in Table 3.

**Figure 1:**
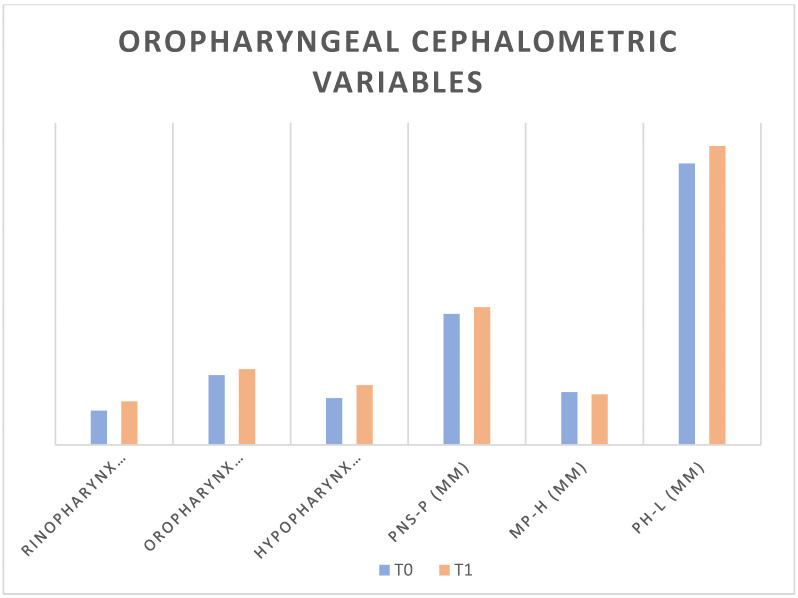
Comparison of the oropharyngeal cephalometric variables at T0 and T1.

Several changes in the pharyngeal measurements were also found. At time T0, the median value of the width of the nasopharynx was 7.5 mm (IQR 6–11), while after treatment, it increased to 9.5 mm (IQR 8.4–14, *p* ≤ 0.05.) The median value of the oropharyngeal dimension at time T0 was 15.2 mm (IQR 14.3–18), while in T1 it increased to 16.65 mm (IQR 15–19, *p* ≤ 0.05.) The hypopharyngeal dimension was also slightly enlarged. The median values relating to the distances PNS-P, MP-H, and PH-L were increased in T1 compared to baseline, with a not statistically significant level (*p* > 0.05). The comparison between the pharyngeal variables measured in T0 and T1 is shown in Table 4. and in Figure 1.

## 4. Discussion

The present investigation analyzed the changes recorded after orthopedic therapy with an RME and a Delaire mask. In the literature, many cephalometric studies have previously evaluated the craniofacial features related to OSAS as predisposing factors in the pathogenesis of upper airway obstruction during sleep [3]. The efficacy of orthopedic treatment with an RME in patients with OSAS was demonstrated in the literature [27,28,29]. The use of the RME as an orthopedic device is confirmed to be effective in promoting the growth of transverse diameters of the maxillary bone. An increase in the nasopharyngeal and oropharyngeal spaces confirms that treatment with a rapid palatal expander is effective in improving the patency of the upper airways [30,31,32]. The association between OSAS and class III malocclusion is an infrequent clinical condition. For this reason, there are no validated therapeutic protocols as yet. Orthopedic treatment with an RME and a Delaire mask is widely used in clinical practice to correct the relationship between the maxillary and mandibular bones [20,21,22]. Among the variety of therapeutic treatments, we chose a protocol for the correction of a Class III malocclusion for the maxilla, thus limiting the approach to Class III cases with maxillary involvement [33,34]. In addition, the positive effects of this treatment on the sagittal pharyngeal dimensions in Class III malocclusion subjects have previously been investigated in the literature [23]. The therapeutic protocol was also chosen in consideration of the fact that mandibular growth has an influence on the size of the upper airways [35,36,37,38,39,40,41,42,43,44,45]. Therefore, it can be hypothesized that jaw growth could also have beneficial effects on the upper airways. Mandibular distraction osteogenesis may also be helpful for treating OSAS in patients with maxillary hypoplasia and severe upper airway obstruction [35].

Based on these findings, the aim of this preliminary study was to confirm the positive effects of an RME and Delaire mask therapy on the upper airway dimensions and evaluate the improvement of OSAS-related clinical conditions. The results obtained from this study showed a significant variation in some of the cephalometric variables analyzed. The increase in SNA angle proves that point A moved anteriorly and suggests a variation in the sagittal position of the maxilla related to the skull base. Bearing in mind that these cephalometric measurements do not usually change in normal conditions, these increments gain importance as they make a contribution to the goals of this therapy; this is one of the attendant effects of the protraction made by the Delaire mask [20,21].

Moreover, no SNB angle decrease was found, as has been described in the study by A.S. Kilinç [23]. Instead, in accordance with M. Rosa‘s study, no significant changes were found in the sagittal position of the mandible in relation to the skull base (ANB and SNB angles) [21]. This suggests that the correction of class III malocclusion has been determined only BY the forward motion of the maxillary bone and not By below e backward rotation of the jaw.

Regarding the dental parameters, as already described by McNamara and Brudon (1993) [23] and Kim et al. (1999) [35], an increase in the 1^MAX angle was found, which indicates greater uprightness of the upper incisors in relation to the bispinal plane [36,37,38].

Contrary to what was reported by McNamara, in the current study, there was an increase in the 1^MAND angle and a decrease in the interincisal angle (T0 = 145°, after treatment, T1 = 131°). These findings suggest a correction of the back inclination of the lower incisors. This is also confirmed by changes to the dentition’s anterior limit: an increase of 1s-A-Po indicates the greater protrusion of the upper incisor in relation to the basal plane, while an increase of 1i-A-Po indicates greater protrusion of the lower incisor in relation to the basal plane. 

Our analysis of airway measurements reported the following results: before treatment, the nasopharyngeal value was 7.5 mm. After the treatment, there was an increase in this value to 9.5 mm. An important result is an increase in oropharyngeal dimension from 15.2 mm, before treatment, to 16.65 mm at the end of the treatment. Therefore, as noted previously by Pavoni et al. [18], significant changes in the parameters related to upper airway patency were observed. A small increase in the median values of the hypopharynx has been noted for the mean values in T0 and T1 of 10.2 mm and 13 mm, respectively, but this occurred with an unequal distribution, and therefore cannot be considered to be statistically significant; also, this value must be considered in relation to the MP-H and PNS-P values [39]. The result was an increase in the median values of the MP-H, PH-L, and PNS-P, which is indicative of a greater distance between the hyoid bone and the mandibular plane and proves an increase in the palate’s sagittal dimensions. As already shown in the literature, these changes are closely related to an improvement in OSAS-related clinical conditions [40,41,42]. In particular, the change in the hyoid bone’s position has a significant impact on the shape and position of the tongue, affecting the patency of the airways [18,19,20,21,22,23,24,25,26,27,28,29,30,31,32,33,34,35,36,37,38,39,40,41,42,43,44]. As reported in other studies, in this study, we found a significant increase in the maxillary forward position. In addition, mandibular forward movement and downward and backward rotation were inhibited; this finding suggests that the most significant changes occurred in the maxillary bone [23]. The use of the Delaire mask for protraction has been proven to be a valid aid in improving nasal breathing [45], which is essential for the correct growth of the palate and the pneumatic development of the maxillary bone and upper airways. These results confirm that a treatment that changes the position of the jaw bones, the tongue, and the soft palate will also have an effect on the oropharyngeal airway dimensions that are closely related to these structures [35,46,47].

### Limitations of the Study

In this preliminary study, there is no control group; therefore, it provides no evidence as to the effectiveness of the treatments in varying the cephalometric parameters. Our study is limited to a two-dimensional assessment. No volume ratings were calculated because three-dimensional diagnostic techniques are not a routine form of examination in the pediatric population. The improvement of the clinical conditions in terms of sleep-related breathing disorders occurred preliminarily through a clinical control examination. No values relating to the AHI index at baseline were reported because, in this ongoing study, we aim to perform further evaluations with a polysomnographic follow-up.

## 5. Conclusions

The current study reports an increase in nasopharyngeal and oropharyngeal spaces. This study’s findings confirm that treatment with a rapid maxillary expander, when associated with a Delaire mask, effectively improves the patency of the upper airway’s sagittal dimensions. Our preliminary results pave the way for further controlled studies in order to confirm the value of this protocol when used as a treatment in pediatric patients with class III malocclusion who are affected by OSAS.

## Figures and Tables

**Table 1:** Cephalometric variables, as measured and analyzed.

**Skeletal Variables**	
SNA (grades)	Angle identified by the points S, N, and A
SNB (grades)	Angle identified by the points S, N, and B
ANB (grades)	Difference between SNA and SNB angles
SN^GOGN (grades)	Angle identified by the intersection of the S-N and GO-GN planes
FM^ (grades)	Angle identified by the intersection of the Frankfurt and mandibular planes
MM^ (grades)	Angle identified by the intersection of the bispinal and mandibular planes
**Dental variables**	
OVB (mm)	Vertical distance between the incisal edge of upper and lower incisor
OVJ (mm)	Horizontal distance between the incisal edge of upper and lower incisor
1^MAX (grades)	Angle identified by the intersection of the axis of the upper incisor and the bispinal plane
1s-N-FH (mm)	Distance between the upper incisor and the N-FH line (Frankfurt plane)
1i-N-FH (mm)	Distance between the lower incisor and the N-FH line (Frankfurt plane)
1i^MAND (grades)	Angle identified by the intersection of the axis of the lower incisor and the mandibular plane
1s-A-Po (mm)	Distance between the upper incisor and the A-Po line
1i-A-Po (mm)	Distance between the lower incisor and the A-Po line
1s^1i (grades)	Angle identified by the intersection of the axes of the upper and lower central incisors
Ul-E-line (mm)	Distance between upper lip (Ul point) and Ricketts’s aesthetic line (tip of nose–tip of chin)
Ll-E-line (mm)	Distance between upper lip (Ll point) and Ricketts’s aesthetic line (tip of nose–tip of chin)
**Upper airway space** **dimensions**	
Nasopharynx (mm)	Distance from a point on the posterior contour of the soft palate to the point closest to the posterior wall of the pharynx
Oropharynx (mm)	Distance from the intersection of the posterior edge of the tongue and the lower edge of the mandible to the point closest to the posterior wall of the pharynx
Hypopharynx (mm)	Distance from a point on the posterior base of the tongue to the point closest to the posterior wall of the pharynx
PNS-P (mm)	Distance between the lowest point of the soft palate (P) and the posterior nasal spine (PNS)
MP-H (mm)	Distance between the mandibular plane (MP) and the hyoid bone (H)
PH L (mm)	Distance between the base of the epiglottis (Eb) and the tip of the tongue (Tt)

Linear measurements are expressed in millimeters (mm); angular measurements (^) are expressed in degrees.

**Table 2:** Descriptive characteristics of the sample (*N* = 14).

**Gender, n (%)**	Male Female	6 (42.86) 8 (57.14)
**Age, median (IQR)**		8 (7–9)
**Clinical conditions associated with respiratory sleep disorders, n (%)**		
Apnea	No Yes	5 (35.71) 9 (64.29)
Rhinitis	No Yes	7 (50.00) 7 (50.00)
Roncopathy	No Yes	12 (85.71) 2 (14.29)
Andenotonsillar hypertrophy	No Yes	6 (42.86) 8 (57.14)
Allergy	No Yes	7 (50.00) 7 (50.00)
**Mixed dentition, n (%)**	No Yes	1 (7.14) 13 (92.86)

Variables were expressed as median and interquartile ranges (IQRs).

**Table 3:** Comparison of the dental and skeletal cephalometric variables at T0 and T1, expressed as median values and IQRs.

	T0 (IQR)	T1 (IQR)	*p*-Value ^#^
SNA (degree)	78.5 (77 81)	81.5 (78 87)	0.041 *
SNB (degree)	77 (76 82)	77.5 (75 83)	0.298
ANB (degree)	2.5 (1 5)	3 (2 4)	0.430
SN^GOGN (degree)	35 (32 38)	34.5 (30 38)	0.173
FM^ (degree)	28 (25 30)	27.5 (24 30)	0.063
MM^ (degree)	27.5 (26.5 32)	29 (27 32)	0.395
1^MAX (degree)	101 (95 109)	114 (104 116)	0.016 *
1s-N-FH (mm)	41.15 (37.4 43.5)	44.75 (42 52.5)	0.014 *
1i-N-FH (mm)	39.5 (37 43)	45 (41 49.5)	0.033 *
1i^MAND (degree)	84.5 (81 89)	90 (87 94)	0.019 *
1s-A-Po (mm)	1.85 (0.2 4.5)	5.5 (2.2 17)	0.028 *
1i-A-Po (mm)	1.9 (1.1 2)	2.15 (2 11)	0.027 *
1s^1i (degree)	145 (139 154)	131 (123 136)	0.020 *
Ls-Le (mm)	2.25 (0 3.9)	1.25 (−2 2)	0.637
Li-Le (mm)	1.5 (0 2.8)	0 (−1.5 1)	0.429
OVB (mm)	1.4 (0 3)	0.85 (0.1 1.5)	0.615
OVJ (mm)	−0.5 (−1.32 3)	2.41 (0.3 3)	0.109

^#^ Wilcoxon signed-rank test. * statistical significance, (*p* ≤ 0.05). Linear measurements are expressed in millimeters (mm); angular measurements (^) are expressed in degrees.

**Table 4:** Comparison of the oropharyngeal cephalometric variables at T0 and T1, expressed as median values and IQRs.

	T0 (IQR)	T1 (IQR)	*p*-Value ^#^
Rinopharynx (mm)	7.5 (6 11)	9.5 (8.4 14)	0.009 *
Oropharynx (mm)	15.2 (14.3 18)	16.65 (15 19)	0.020 *
Hypopharynx (mm)	10.2 (8 14)	13 (10 16)	0.095
PNS-P (mm)	28.5 (25 32)	30 (26 30)	0.875
MP-H (mm)	11.5 (9.35 15)	11 (7.8 19)	0.530
PH-L (mm)	61.2 (54 66)	65 (56.3 70)	0.258

^#^ Wilcoxon signed-rank test. * Statistical significance (*p* ≤ 0.05). Linear measurements are expressed in millimeters (mm).
